# Supplementary material for: Differential Expression of Mitochondrial Biogenesis Markers in Mouse and Human SHH-Subtype Medulloblastoma
Source: Cells. 2019 Mar 5;8(3):216. doi: 10.3390/cells8030216 (PMC6468894; doi:10.3390/cells8030216)
Supplement: Supplementary file 1 [file cells-08-00216-s001.zip › Supplemental figures.docx]

**Supplemental figures**


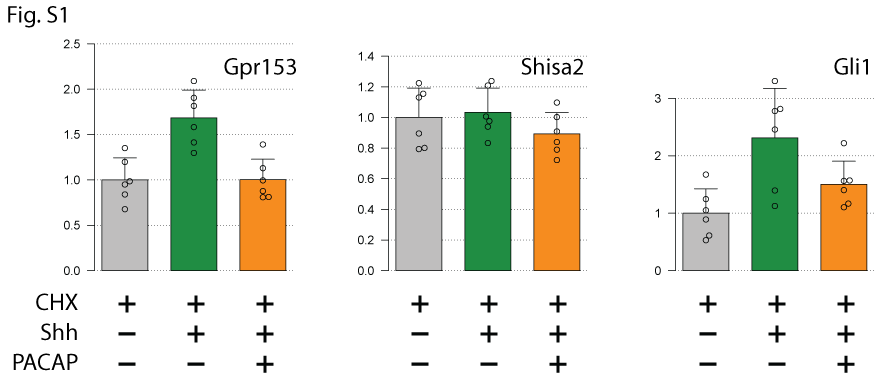


**Figure S1.** Upregulation of Shisa2, but not Gpr153 or Gli1, is dependent on new protein synthesis. (A) Cultured cGCPs were pre-treated with 10μg/mL cycloheximide (CHX) and then treated for 6h with 1μg/mL Shh-N and/or 10nM PACAP38 in the presence of CHX. Shown is the expression of indicated genes by RT-qPCR (mean +/- SD) from n=6 independent samples/group.


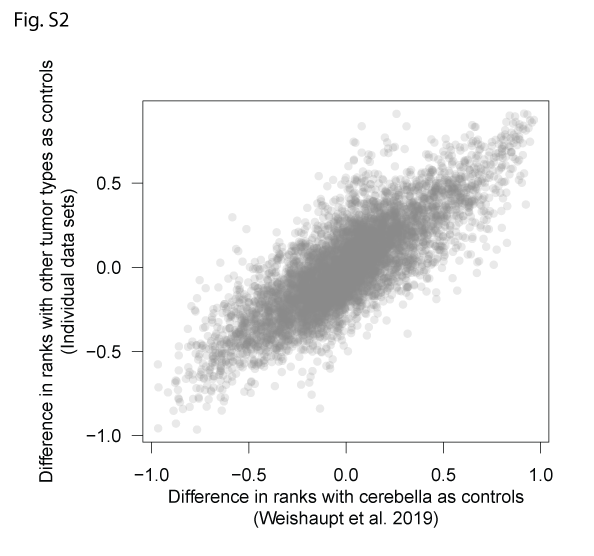


**Figure S2.** Comparison between gene rank differences (human vs. mouse) that result from analyses that use healthy cerebellum tissues as controls (human dataset from Weishaupt et al., 2019; x-axis) and analyses that use non-SHH MB as controls in human (y-axis). Each data point corresponds to a single gene. Gene rank differences are very similar irrespective of controls used.


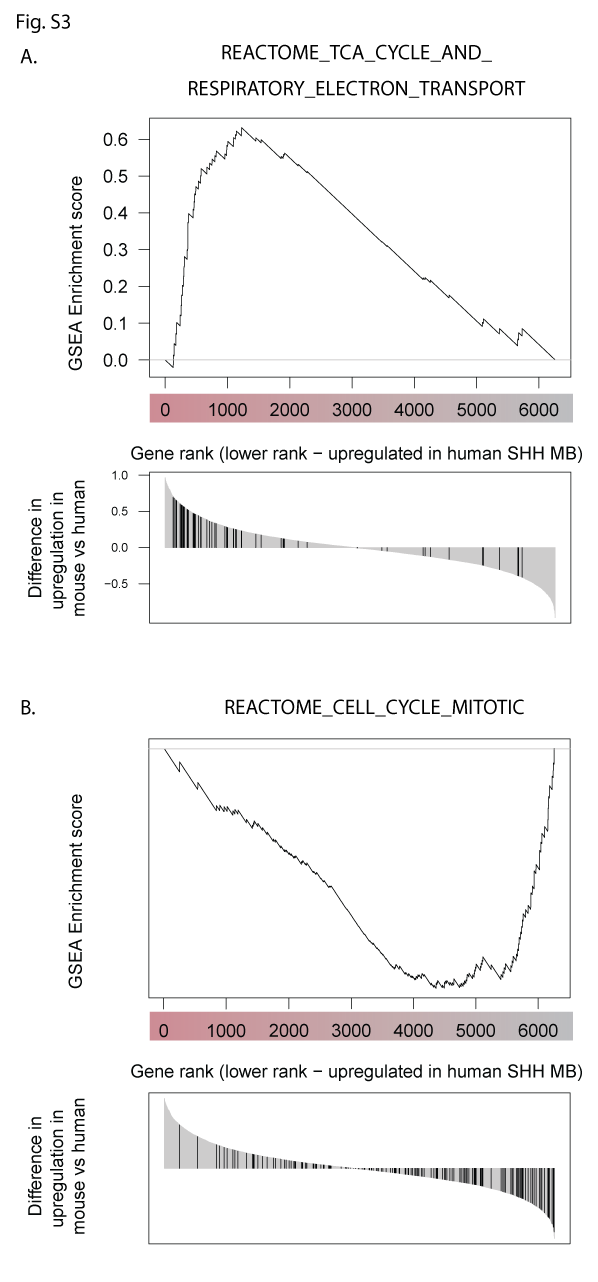


**Figure S3.** GSEA plots for the indicated gene sets generated from gene ranks calculated using healthy cerebella as controls for both human and mouse datasets. The plots look very similar to respective plots generated from gene ranks using non-SHH MB as controls in human (see Fig. 5A, B).
